# Connaissances et pratiques des étudiants en fin d’études de pharmacie à l’Université Cheikh Anta Diop sur l’utilisation des antibiotiques et la résistance bactérienne en 2019 (Sénégal)

**DOI:** 10.11604/pamj.2023.44.127.28905

**Published:** 2023-03-14

**Authors:** Oumar Bassoum, Ndèye Marème Sougou, Ousmane Djiby Ndiaye, Makhtar Camara, Djibril Fall

**Affiliations:** 1 Service de Médecine Préventive et Santé Publique, Faculté de Médecine, de Pharmacie et d’Odontologie, Université Cheikh Anta Diop, Dakar, Sénégal,; 2 Institut de Santé et Développement, Université Cheikh Anta Diop, Dakar, Sénégal,; 3 Faculté de Médecine, de Pharmacie et d´Odontologie, Université Cheikh Anta Diop, Dakar, Sénégal,; 4 Laboratoire de Bactériologie-Virologie, Centre Hospitalier National Universitaire Aristide Le Dantec, Faculté de Médecine, de Pharmacie et d´Odontologie, Université Cheikh Anta Diop, Dakar, Sénégal,; 5 Institut de Recherche en Santé de Surveillance Epidémiologique et de Formation, Dakar, Sénégal,; 6 Laboratoire de Chimie Thérapeutique et Organique, Faculté de Médecine, de Pharmacie et d'Odontologie, Université Cheikh Anta Diop, Dakar, Sénégal,; 7 Laboratoire National de Contrôle des Médicaments, Dakar, Sénégal

**Keywords:** Antibiotiques, connaissances, pratiques, étudiants en pharmacie, Sénégal, Antibiotics, knowledge, practice, pharmacy students, Senegal

## Abstract

**Introduction:**

les étudiants en pharmacie sont parmi les futurs professionnels de la santé qui devraient jouer un rôle essentiel dans la lutte contre la résistance bactérienne (RB). L´objectif de notre travail était d´étudier les connaissances et pratiques des étudiants en fin d´études de pharmacie sur la RB et l´utilisation des antibiotiques.

**Méthodes:**

l´étude était transversale, descriptive et analytique. La population d´étude était constituée des étudiants inscrits en master 2 et en doctorat de pharmacie de l´Université Cheikh Anta Diop durant l´année 2019. Les données étaient collectées entre juillet et octobre 2019 au moyen d´un questionnaire électronique dont le lien était partagé à travers le réseau social WhatsApp. Les connaissances étaient évaluées avec une échelle de Likert à cinq niveaux tandis que des questions fermées étaient utilisées pour déterminer les pratiques. Les analyses descriptives ont été réalisées. Les facteurs associés aux pratiques étaient identifiés à l´aide de la régression logistique. Les analyses sont réalisées avec le logiciel Epi Info™ 7.2.2.16. Le seuil de signification était fixé à 0,05.

**Résultats:**

sur les 559 étudiants éligibles, 278 ont répondu au questionnaire, soit un taux de participation de 60,6%. Parmi eux, 72,3% ont déclaré avoir consommé des antibiotiques durant les 12 mois précédant l´enquête. Concernant les connaissances, 85,6% des étudiants interrogés avaient un niveau suffisant. En outre, 38,2% des étudiants avaient des pratiques inadéquates. Celles-ci étaient associées au fait d´avoir un parent ou un ami comme agent de santé (OR = 1,69; p-value = 0,04), d´être en doctorat (OR = 0,55; p-value = 0,02) et d´avoir un niveau de connaissances insuffisant (OR = 2,21; p-value = 0,02).

**Conclusion:**

cette étude a montré que la consommation des antibiotiques est élevée chez les étudiants en pharmacie et que leurs pratiques étaient inadéquates malgré le niveau satisfaisant de leurs connaissances sur les antibiotiques et la RB. Il urge de renforcer la sensibilisation des étudiants et de leur entourage sur les bonnes pratiques d´utilisation des antibiotiques.

## Abstract (en)

**Introduction:**

pharmacy students are future providers of pharmaceutical care and should play a critical role in combating bacterial resistance (BR). The purpose of this study was to evaluate the knowledge and practice of students at the end of pharmacy course relating to BR and antibiotic use.

**Methods:**

we conducted a cross-sectional, descriptive and analytic study. The study population consisted of students enrolled in Master 2 and PhD in Pharmacy at the Cheikh Anta Diop University over the year 2019. Data were collected between July and October 2019 using an electronic questionnaire whose link was shared through the social network WhatsApp. Knowledge was assessed using a 5-point Likert scale while closed-ended questions were used to determine practice. Descriptive analyses were performed. Factors associated with practice were identified using logistic regression. Analyses were performed using the EPI Info^TM^ software 7.2.2.16. The significance threshold was set to 0.05.

**Results:**

out of 559 eligible students, 278 responded to the questionnaire, reflecting a participation rate of 60.6%. Of these, 72.3% reported having used antibiotics in the 12 months preceding the survey. Regarding knowledge, 85.6% of students surveyed had an adequate level. In addition, 38.2% of students had inadequate practice. These were associated with having a relative or friend as a health worker (OR = 1.69; p-value = 0.04), being a PhD student (OR = 0.55; p-value = 0.02) and having an insufficient level of knowledge (OR = 2.21; p-value = 0.02).

**Conclusion:**

this study shows that antibiotic consumption is high among pharmacy students and that their practice is inadequate despite their satisfactory level of knowledge about antibiotics and BR. It is urgent to strengthen the awareness of students and their entourage about good practices concerning antibiotic use.

## Introduction

A l´échelle mondiale, une part importante de la morbidité et de la mortalité est liée aux maladies bactériennes [1]. Cette situation a stimulé la recherche ayant abouti à la découverte des antibiotiques [2]. Ces derniers ont révolutionné la médecine et permis de sauver plusieurs vies humaines [3]. Toutefois, le monde fait face à l´émergence et à la diffusion massive de la résistance bacterienne (RB) [2]. Celle-ci correspond au fait qu´un traitement antibiotique ne soit plus efficace sur une infection bactérienne [4]. Ce phénomène représente l´une des plus graves menaces pour la santé mondiale, la sécurité alimentaire et le développement. Toute personne, quels que soient son âge et son pays, peut etre touchée [5]. D´après le rapport du système mondial de surveillance de la résistance aux antimicrobiens (RAM), les taux de résistance aux antibiotiques les plus couramment utilisés sont élevés. Par exemple, le taux de résistance à la ciprofloxacine variait de 8,4% à 92,9% pour *Escherichia coli* et de 4,1% à 79,4% pour *Klebsiella pneumoniae* [6]. Au Sénégal, des cas de résistance ont été rapportés. Par exemple, une étude portant sur des échantillons collectés entre janvier et décembre 2016 avait indiqué que les prévalences des entérobactéries productrices de bêta-lactamases à spectre élargi (BLSE) et de carbapénèmases étaient respectivement estimées à 26,2% et 5,1% [7]. Une autre étude avait évalué le profil de sensibilité de 20 souches de *Haemophilus influenzae* collectées entre mai et novembre 2014. Les résultats avaient révélé qu´elles étaient résistantes à l´association triméthoprime/sulfaméthoxazole [8].

Les causes de la RB sont, entre autres, la dispensation et la prescription inappropriées, l´automédication, l´utilisation execessive des antibiotiques en médecine vétérinaire et dans l´agriculture et le faible nombre de nouveaux antibiotiques mis sur le marché [3,9]. Les conséquences sont alarmantes. Un nombre croissant d´infections, comme la pneumonie, la tuberculose, la gonorrhée et la salmonellose, deviennent plus difficiles à traiter. La prolongation des hospitalisations, l´augmentation des dépenses médicales et la hausse de la mortalité deviennent courantes [5]. De même, la RB compromet la réalisation de certaines procédures médicales telles que la transplantation, l'intubation, la chirurgie lourde ou la chimiothérapie anticancéreuse, qui nécessitent l'utilisation d'antibiotiques pour prévenir ou traiter l'infection associée [10]. Compte tenu de l´ampleur de la RB et de ses conséquences, l´Organisarion Mondiale de la Santé (OMS) a élaboré le « Plan d'action mondial pour combattre la RAM ». Ce plan a défini cinq objectifs dont l´un consiste à mieux faire connaître et comprendre le problème de la RAM grâce à une communication, une éducation et une formation efficaces. Les étudiants en pharmacie constutent une cible importante devant bénéficier d´une formation solide afin de les préparer à entrer dans la vie active avec des compétences et attitudes adéquates pour contribuer à la lutte contre la RAM [11]. C´est dans ce contexte qu´il est apparu nécessaire d´évaluer les connnaissances et le comportement des étudiants en pharmacie sur la RB et l´utilisation des antibiotiques. Plusieurs études se sont déjà interessées à cette question. En Malaisie, au Trinité-et-Tobago, au Royaumes-Unis et au Sri Lanka, des études transversales ont respectivement mis en évidence un bon niveau de connaissances [12-15]. En Revanche, au Rwanda une enquête a révélé un niveau de connaissances moyen [16], alors que les attitudes étaient inadéquates dans d´autres pays [12,13]. En outre, une étude conduite auprès d´étudiants en pharmacie issues respectivement de trois universités d´Indonésie, du Paskistan et de la Malaisie a mis en lumière des lacunes en termes de connaissances sur les antibiotiques [17].

Au Sénégal, l´évaluation externe conjointe conduite par l´OMS dans le cadre du Règlement Sanitaire International a montré deux insuffisances majeures. La première est la faiblesse de la surveillance des infections causées par des agents pathogènes résistants aux antimicrobiens. La deuxième insuffisance concerne l´utilisation irrationnelle des antibiotiques caracterisée par la mauvaise prescription, l´automédication, le marché illicite et la contrefaçon. Des études sont réalisées auprès du grand pubic [18] et des pharmaciens d´officine en exercice [19]. Les résultats font état de lacunes dans les connaissances et les pratiques en matière d´antibiotiques. En revanche, aucune enquête de ce genre ne s´est intéressée aux étudiants en pharmacie. C´est sur la base de ce constat que cette étude tente d´explorer les questions de recherche suivantes: 1) quel est le niveau de connaissances des étudiants en fin d´études de pharmacie en matière de RB et d´utilisation des antibiotiques? 2) Quelles sont les pratiques des étudiants en fin d´études de pharmacie en matière d´utilisation des antibiotiques? 3) Quels sont les facteurs influençant les pratiques des étudiants en fin d´études de pharmacie en matière d´utilisation des antibiotiques? Les réponses à ces questions comportent un double intérêt. Le premier est que cette étude permettra de comprendre le niveau de prise de conscience des étudiants vis-à-vis de la RB, et par conséquent contribuer à la mise en place de stratégies de lutte contre les mauvaises pratiques. Le deuxième intérêt est académique en ce sens que l´étude représente une occasion de renforcer le programme de formation des étudiants en matière de RB et d´utilisation des antibiotiques. L´objectif de cette étude était de mesurer le niveau de connaissances et de déterminer les pratiques des étudiants en fin d´études de pharmacie à l´Université Cheikh Anta Diop (UCAD) sur la RB et l´utilisation des antibiotiques.

### Cadre d´étude

Cette étude avait pour cadre la Faculté de Médecine, de Pharmacie et d´Odontologie de l´UCAD. Cette faculté comporte trois sections: médecine, pharmacie et odontologie. La section de pharmacie est subdivisée en deux départements: le Département des Sciences Pharmaceutiques, Physiques et Chimiques et le Département des Sciences Biologiques et Pharmaceutiques Appliquées [20]. La faculté a adopté le système LMD, avec L pour licence, M pour master et D pour Doctorat. La formation est orgnisée en deux semestres dont chacun est constitué d´unités d´enseignement (UE). Chaque UE est composée d´éléments constitutifs (EC). Les étudiants en pharmacie bénéficient d´enseignements relatifs à la bactériologie et à l´utilisation des médicaments dont le contenu varie d´un niveau à l´autre. La bactériologie et la virologie sont enseignées en deuxième et troisième années de licence et en premiére année de master (option biologie). Les cours de chimie thérapeutique et de pharmacologie sont donnés en troisième année de licence et en première année de master. La pharmaco-vigilance et la pharmacie clinique sont enseignées en deuxième année de master [21].

## Méthodes

**Type et période d´étude:** l´étude était transversale, descriptive et analytique. Les données étaient collectées entre juillet et octobre 2019.

**Population d´étude:** la population cible était constituée des étudiants inscrits en master 2 et en doctorat de pharmacie à l´UCAD en 2019. La population source était constituée des étudiants membres des deux groupes WhatsApp appartenant respectivement aux deux promotions.

**Échantillonnage:** au moment de l´enquête, les groupes WhatsApp des deux promotions comptaient respectivement 235 et 224 membres, soit 459 membres. Ceux-ci étaient tous invités à participer à l´enquête.

### Collecte des données

**Outil de collecte:** un questionnaire était élaboré sur la base de la revue de la littérature [22,23] et adapté au contexte du Sénégal. L´application Google Forms était utilisée pour la confection d´un questionnaire électronique. Les thématiques abordées étaient les suivantes: 1) les caractéristiques socio-démographiques; 2) la fréquence de consommation des antibiotiques; 3) les connaissances sur les antibiotiques et la RB (identification, indications, effets secondaires, résistance); 4) la sensibilisation sur la RB; 5) les pratiques d´utilisation des antibiotiques.

**Méthode de collecte:** la technique de collecte était une auto-administration qui consistait à remplir le questionnaire électronique dont le lien était transmis aux étudiants à travers leurs deux groupes WhatsApp. Les délégués des deux promotions étaient sollicités pour obtenir un fort taux de participation. Des relances étaient émises à la fin de chaque semaine.

**Variables collectées:** les six variables socio-démographiques collectées étaient le niveau d´étude (master 2/doctorat), l´âge (variable quantitative), le sexe (masculin/féminin), l´existence d´un agent de santé dans son entourage (parent ou ami) (oui/non) et le statut matrimonial (marié/non marié). La consommation des antibiotiques au cours des 12 mois ayant précédé l´enquête (oui/non) était collectée. Ensuite, la fréquence de la consommation était recueillie (1-2 fois, 3-5 fois, > 5 fois). Les connaissances étaient évaluées à l´aide de 14 questions à échelle de Likert. Les questions étaient formulées sous forme de phrases déclaratives. Les modalités de réponse étaient 'accord total', 'accord partiel', 'neutre', 'désaccord partiel' et 'désaccord total'. Le score variait de 1 à 5 points. Lorsque la déclaration était vraie, 'accord total' était côté 5 et 'désaccord total' était côté 1. En revanche, lorsque la déclaration était fausse, 'accord total' était côté 1 et 'désaccord total' était côté 5. Toutes les déclarations étaient vraies sauf les déclarations 2, 3, 5, 6 et 14. Les scores minimal et maximal attendus étaient respectivement 14 et 70 points. La sensibilisation portait sur le fait d´avoir entendu parler de la RB (oui/non), le fait d´avoir discuté de la RB durant les cours de pharmacie (oui/non) et les sources d´information autres que les cours de pharmacie (questions à choix multiples). La variable relative aux pratiques était collectée à l´aide de 7 questions fermées (oui/non).

**Définitions opérationnelles des variables:** deux niveaux de connaissances étaient définis: 1) (14-56): connaissances insuffisantes; 2) (57-70): connaissances suffisantes. Deux niveaux de pratiques étaient définis: 1) pratiques inadéquates 0-4; 2) pratiques adéquates 5-7.

**Analyses statistiques:** les données étaient importées vers un fichier Excel et analysées à l´aide de Epi Info^TM^ 7.2.2.16 qui est un logiciel développé par les centres américains pour le contrôle et la prévention des maladies. Les variables quantitatives étaient décrites à l´aide des paramètres de position et de dispersion. Les variables qualitatives étaient exprimées sous forme d´effectif et de pourcentage. Le test du Chi^2^ de Pearson et la régression logistique étaient réalisés pour identifier les facteurs associés aux pratiques des étudiants en matière d´utilisation des antibiotiques. La variable dépendante était représentée par les pratiques inadéquates (oui/non). Les variables indépendantes étaient les caractéristiques socio-démographiques, la fréquence de la consommation d´antibiotiques et les connaissances. Le critère de sélection des variables d´entrée dans la régression logistique était une p-value inférieure ou égale à 0,25 [24]. Les odds ratio (OR) étaient calculés et entourés de leur intervalle de confiance à 95%. Une différence statistiquement significative était établie lorsque la p-value est inférieure ou égale à 0,05.

**Considérations éthiques:** une lettre d´information accompagnait le questionnaire et précisait l´objectif de l´enquête, les résultats attendus ainsi que la nature confidentielle des données. Les étudiants étaient informés que l´étude ne rentrait pas dans le cadre des évaluations et que leur refus de participer n´entrainait pas de sanction. Seule l´équipe de recherche avait accès aux données.

## Résultats

**Taux de participation:** parmi les 459 étudiants éligibles, 278 ont répondu au questionnaire soit un taux participation de 60,6%.

**Caractéristiques sociodémographiques:** l´âge médian était estimé à 26 ans. L´intervalle interquartile se situait entre 25 et 27 ans. Les étudiants âgés d´au moins 26 ans étaient majoritaires (58,3%). Les hommes représentaient 56,8%. Les mariés étaient de 15,8%. Les participants ayant déclaré avoir un proche ou un membre de leur entourage qui travaille dans le domaine de la santé représentaient 50,7%. Les étudiants inscrits en doctorat représentaient 63% (Tableau 1).

**Tableau 1:** caractéristiques socio-démographiques des répondants (N = 278)

Caractéristiques socio-démographiques	Effectif	Pourcentage (%)
**Classe d'âge (ans)**		
<26 ans	116	41,7
≥26 ans	162	58,3
**Sexe**		
Masculin	158	56,8
Féminin	120	43,2
**Statut marital**		
Marié	44	15,8
Non marié	234	84,2
**Existence d'un agent de santé dans son entourage (parent ou ami)**		
Oui	141	50,7
Non	137	49,3
**Niveau d'étude**		
Master 2	103	37
Doctorat	175	63

**Consommation des antibiotiques:** selon l´étude, 72,3% (201/278) des étudiants ont déclaré avoir pris un traitement antibiotique au cours des 12 mois ayant précédé l´enquête. Parmi eux, 77,11% (155/201) ont déclaré avoir consommé des antibiotiques 1 à 2 fois durant l´année (Tableau 2).

**Tableau 2:** répartition des répondants selon la fréquence de prise des antibiotiques (N = 201)

Nombre de prises	Effectif	Pourcentage (%)
1-2	155	77,11
3-5	32	15,92
Supérieur à 5	14	6,97
Total	201	100

**Connaissances:** tous les étudiants interrogés reconnaissaient que les pénicillines sont des antibiotiques et que le paracétamol et l´aspirine ne le sont pas. Concernant l´indication, 90,6% des répondants ont mentionné leur accord total quant à l´utilité des antibiotiques contre les infections bactériennes. En outre, 63,7% ont indiqué leur désaccord total au sujet de l´utilité des antibiotiques contre les infections virales alors que 20,9% pensaient le contraire. L´étude a également montré que 78,4% ont affiché leur accord total sur l´indication des antibiotiques pour tout type de douleur et d´inflammation. Quant aux effets secondaires, des répondants étaient totalement d´accord que les antibiotiques peuvent tuer les “bonnes bactéries” présentes dans notre organisme (51,4%), causer des infections secondaires après avoir tué les “bonnes bactéries” présentes dans notre organisme (48,9%) et causer des réactions allergiques (76,6%).

Par ailleurs, 56,5% et 49,6% des étudiants interrogés ont respectivement fait part de leur accord total sur le fait que l´utilisation des antibiotiques dans l´élevage et dans l´agriculture peut être néfaste pour la santé humaine. En outre, 87,1% des répondants étaient totalement d´accord que la résistance aux antibiotiques est un phénomène durant lequel une bactérie devient insensible à un antibiotique. De même, 84,9% étaient totalement d´accord que la mauvaise utilisation d´un antibiotique peut entrainer une RB. Dans 89,2% des cas, les étudiants étaient totalement conscients qu´on ne pas peut interrompre un traitement antibiotique même si les symptômes s´améliorent avant que l´on termine la quantité prescrite (Tableau 3).

**Tableau 3:** connaissances des répondants sur la RB et l’utilisation des antibiotiques (N = 278)

Affirmations	*DT	*DP	*N	*AP	*AT
	%	%	%	%	%
Les pénicillines sont des antibiotiques	-	-	-	-	100
L'aspirine est un antibiotique**	100	-	-	-	-
Le paracétamol est un antibiotique**	100	-	-	-	-
Les antibiotiques sont utiles contre les infections bactériennes	-	-	-	9,4	90,6
Les antibiotiques sont utiles contre les infections virales**	63,7	12,6	2,9	16,2	4,7
Les antibiotiques sont indiqués pour réduire tout type de douleur et d'inflammation**	78,4	10,8	2,2	7,9	0,7
Les antibiotiques peuvent tuer les “bonnes bactéries” présentes dans notre organisme	3,6	7,9	6,5	30,6	51,4
Les antibiotiques peuvent causer des infections secondaires après avoir tué de bonnes bactéries présentes dans notre organisme	8,6	4	9	29,5	48,9
Les antibiotiques peuvent causer des réactions allergiques	1,4	0,4	1,4	20,1	76,6
L'utilisation des antibiotiques dans l'élevage peut être néfaste pour la santé humaine	2,5	4	9,4	27,7	56,5
L'utilisation des antibiotiques dans l'agriculture peut être néfaste pour la santé humaine	4	3,6	14	28,1	49,6
La résistance aux antibiotiques est un phénomène lors duquel une bactérie perd sa sensibilité à un antibiotique	3,6	0,7	0,7	7,9	87,1
La mauvaise utilisation d'un antibiotique peut entrainer une perte de sa sensibilité à un agent pathogène précis	0,7	0,4	3,2	10,8	84,9
On peut interrompre un traitement antibiotique lorsque les symptômes s'améliorent avant que l'on termine de prendre le traitement complet**	89,2	4	2,2	2,2	2,5

**Classification du niveau des connaissances:** le score moyen des connaissances sur les antibiotiques était de 60,8 avec un écart type de 4,1. Le score minimal était égal à 46 tandis que le score minimal était de 70. La médiane était de 62. Le niveau de connaissances de 85,6% (238/278) des répondants était jugé suffisant tandis que celui de 14,4% (40/278) était considéré comme insuffisant.

**Figure 1:**
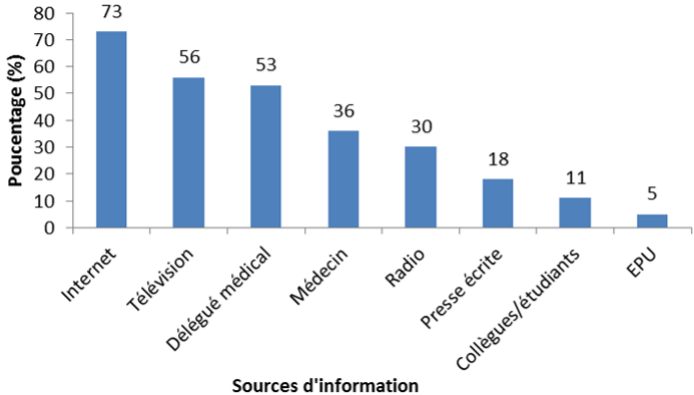
répartition des répondants suivant les sources d’information au sujet de la RB (N = 256)

**Sources d´information:** tous les étudiants interrogés ont déclaré avoir déjà entendu parler de la RB lors des cours de pharmacie. Cependant, 92% des participants (256/278) ont affirmé avoir entendu parler de ce sujet à travers des canaux autres que les cours de pharmacie. Les répondants ont notamment cité l´internet (73%), la télévision (56%), le délégué médical (53%) et le médecin (36%) (Figure 1).

**Pratiques:** l´étude a révélé que 42,9% des étudiants interrogés ont dit prendre des antibiotiques pour traiter le rhume ou l´angine. De même, 19,8% des répondants ont déclaré interrompre leur traitement antibiotique lorsqu´ils commencent à se sentir mieux. La conservation des restes d´antibiotiques à la maison était une pratique déclarée par 35,6% des répondants. Près de 7 étudiants sur 10, soit 69,1% ont déclaré acheter des antibiotiques sans prescription médicale (Tableau 4).

**Tableau 4:** pratiques des répondants en matière d’utilisation des antibiotiques (N = 278)

Questions	Oui	%
Prenez-vous habituellement des antibiotiques contre le rhume ou l'angine ?	117	42,9
Prenez-vous habituellement des antibiotiques contre la fièvre ?	15	5,4
Arrêtez-vous habituellement de prendre des antibiotiques lorsque vous commencez à vous sentir mieux ?	55	19,8
Conservez-vous les restes d'antibiotiques à la maison pour les réutiliser à l'avenir ?	99	35,6
Utilisez-vous les restes d'antibiotiques lorsque vous avez le rhume, l'angine ou la grippe sans consulter le médecin ?	70	25,2
Achetez-vous des antibiotiques sans prescription médicale ?	192	69,1
Avez-vous déjà commencé un traitement antibiotique après un simple appel téléphonique avec un médecin, sans un examen médical adéquat ?	115	41,4

**Classification des pratiques des répondants en matière d´utilisation des antibiotiques:** l´étude a révélé que les pratiques de certains participants (61,2%) étaient adéquates tandis que celles des autres (38,2%) étaient inadéquates.

**Facteurs associés aux pratiques inadéquates en matière d´utilisation des antibiotiques:** l´étude a indiqué que le sexe et les pratiques en matière d´utilisation des antibiotiques n´étaient pas liés. En revanche, le fait d´avoir un parent ou un ami comme agent de santé multipliait par 1,69 le risque de se livrer aux pratiques inadéquates (p-value = 0,04). De même, les étudiants en Master 2 avaient un risque de s´adonner aux pratiques inadéquates inférieur de 45% á celui observé chez les étudiants en doctorat (OR = 0,55; p-value = 0,02). Le faible niveau de connaissances s´est aussi révélé être un facteur prédisposant aux pratiques inadéquates (OR = 2,21, p-value = 0,02) (Tableau 5).

**Tableau 5:** facteurs associés aux pratiques inadéquates en matière d’utilisation des antibiotiques (N = 278)

Variables	OR brute [IC 95%]	OR ajusté [IC 95%]	P-value
**Sexe**			
Féminin	1,49 [0,92-2,40]	1,47 [0,90-2,42]	0,12
Masculin	Référence	Référence	
**Existence d'un agent de santé dans son entourage (parent ou ami)**			
Oui	1,67 [1,03-2,70]	1,69 [1,03-2,77]	0,04
Non	Référence	Référence	
**Niveau d'étude**			
Master 2	0,60 [0,36-0,98]	0,55 [0,33-0,93]	0,02
Doctorat	Référence	Référence	
**Connaissances**			
Insuffisantes	2,00 [1,02-3,94]	2,21 [1,10-4,44]	0,02
Suffisantes	Référence	Référence	

## Discussion

### Consommation d´antibiotiques

Cette étude a montré que 72,3% des répondants ont pris un traitement antibiotique au cours des 12 mois ayant précédé l´enquête. Une étude sri-lankaise menée en 2016 auprès d´étudiants en pharmacie avait mis en évidence une proportion comparable de 75% [15]. En 2017, une étude conduite en population générale dans une ville sénégalaise a également trouvé le même résultat [25]. En 2013, une enquête avait indiqué une proportion de 45,6% parmi les étudiants en sciences de la santé d´une école de médecine en Italie [23]. Ces résultats montrent que la consommation des antibiotiques est excessive.

### Connaissances

Concernant les connaissances, cette étude a révélé que le niveau des étudiants en pharmacie était satisfaisant dans la plupart des cas. Des résultats similaires étaient mis en évidence chez leurs homologues du Royaume-Uni en 2016 [14], de la Malaisie en 2013 [26] et de l´Inde en 2018 [22]. En revanche, des études publiées en 2019 et réalisées en Irak, au Nigéria et en Arabie Saoudite ont révélé un faible niveau de connaissances des étudiants dans ce domaine [27-29]. Dans cette présente étude, certains étudiants pensaient à tort que les antibiotiques sont utiles contre les infections virales, ne peuvent pas tuer les bactéries commensales et ne peuvent pas causer d´infections à la suite de la destruction des bactéries commensales. Ces données suggèrent qu´il convient d´insister sur l´indication et l´effet délétère des antibiotiques à large spectre sur la flore commensale lors des cours de bactériologie et de pharmacologie.

### Sources d´information

En dehors des cours de pharmacie, les principales sources d´information des étudiants au sujet de la RB et de l´utilisation des antibiotiques étaient internet, la télévision, le délégué médical et le médecin. Trois aspects méritent d´être soulignés au vu de ces résultats. D´abord, il faudrait renforcer les campagnes de sensibilisation en diffusant des messages à travers Internet et la télévision. A cet effet, la création d´un site Internet exclusivement dédié aux informations sur les souches résistantes et l´utilisation des antibiotiques s´avère indispensable. La Direction de la Pharmacie et du Médicament et la Direction des Laboratoires pourraient porter l´initiative. Quant à la télévision, elle est disponible chez 55,5% des ménages sénégalais [30]. Ensuite, une source d´information comme le délégué médical devrait appeler une attitude prudente puisque les messages délivrés par celui-ci pourraient ne pas être indépendants. Le troisième aspect réside dans le fait que le médecin soit une des principales sources d´information, ouvrant ainsi une voie à une collaboration étroite entre tous les professionnels de la santé pour un meilleur échange d´informations sur la RB. La radio est peu citée par les répondants alors qu´elle représente un autre canal que l´on retrouve chez 94,1% des ménages sénégalais [31]. Ainsi, il convient de diffuser des messages à travers cet outil de communication. Le recours à la radio de l´UCAD permettrait d´atteindre une large cible qui va au-delà des étudiants en pharmacie.

### Pratiques

Par ailleurs, l´étude a révélé que les pratiques des étudiants en matière d´utilisation des antibiotiques étaient inadéquates. Les répondants ont déclaré avoir habituellement utilisé les antibiotiques contre le rhume, l´angine ou la fièvre. Une pratique similaire était mise en évidence chez des étudiants rwandais lors d´une étude publiée en 2019 [32]. La plupart des étudiants ont dit qu´ils s´adonnent à l´automédication. Cette pratique était constatée chez des étudiants nigérians et rwandais avec des proportions estimées respectivement à 92,2% [28] et 12,1% [32]. Les connaissances des étudiants en pharmacie sur les antibiotiques peuvent expliquer leur recours à l´antibiothérapie sans prescription médicale [28]. D´autres raisons sont également mentionnées dans la littérature. Il s´agit de la perception que la maladie est bénigne et d´une expérience antérieure avec un antibiotique [28]. L´interruption du traitement antibiotique avant la durée requise était rapportée par certains étudiants. Cette pratique était constatée chez des étudiants italiens en pharmacie [23]. Elle est néfaste et pourrait être la raison de la conservation et de l´utilisation des restes d´antibiotiques qui sont des pratiques déclarées par les étudiants.

Ces résultats montrent qu´il serait indispensable de mettre en place un programme de gérance des antibiotiques en insistant sur deux aspects essentiels comme la réglementation et la formation. Premièrement, il convient d´intensifier l´application de la réglementation de la vente des antibiotiques au Sénégal. Deuxièmement, il s´agira d´enseigner aux étudiants les fondamentaux d´un programme de gérance des antibiotiques [33]. En 2010, la Faculté de Médecine de l'Université de Zambie avait révisé son programme de formation. Les thèmes de la RAM et de l'utilisation rationnelle des médicaments y ont été mis en exergue. L'objectif était que les diplômés entrent dans la vie professionnelle avec les compétences et attitudes leur permettant de s´impliquer activement dans la lutte contre la RAM [11]. Ensuite, ce programme devrait être mis en œuvre dans les structures sanitaires et les officines de pharmacie. Cela permettra de limiter les prescriptions et les dispensations irrationnelles.

### Facteurs associés aux pratiques inadéquates

Le faible niveau de connaissances en matière d´antibiotiques est identifié comme un facteur associé aux pratiques inadéquates. Cela prouve encore une fois la nécessité pressante de renforcer les connaissances des étudiants dans ce domaine afin de favoriser un changement de comportement. En outre, les étudiants en doctorat s´adonnaient plus aux pratiques inadéquates que ceux qui sont en master. Ce résultat pourrait s´expliquer par le fait que les étudiants semblent être plus confiants avec leurs connaissances une fois qu'ils atteignent leur dernière année d´étude, se traduisant par un risque plus élevé de pratiquer l´automédication [23]. Enfin, l´étude a montré que le fait d´avoir un proche travaillant dans le domaine de la santé était un facteur lié aux pratiques inadéquates. En Italie, ce facteur était associé à la conservation des restes d’antibiotiques à domicile et à l´automédication [23].

### Forces et faiblesses de l´étude

Cette étude est la première en son genre au Sénégal. Elle a permis de disposer d´informations pertinentes dont les autorités académiques pourraient se servir pour renforcer l´enseignement de la RB et de l´utilisation des antibiotiques tout au long des études de pharmacie. Les réponses comportant un biais d´acceptabilité sociale seraient aussi moins présentes dans les questionnaires en ligne en raison du caractère anonyme de cette méthode [34]. Cependant, cette étude comporte des limites. Le risque de survenue d´un biais de sélection est réel puisque l´enquête ne s´adressait qu´aux étudiants qui sont membres des groupes WhatsApp. Ceux qui éprouvent des difficultés financières pour se connecter pourraient être sous représentés. Il en est de même pour ceux qui ne font pas partie du groupe WhatsApp qui ne peut pas contenir plus de 256 membres [28]. Une autre limite est la nature transversale de l´étude. Or, dans ce type d´étude, il est parfois difficile d´établir la séquence temporelle entre le facteur d´exposition et l´événement puisque ces deux variables sont recueillies en même temps [35]. Par conséquent, des études supplémentaires permettraient de mieux comprendre les déterminants des pratiques des étudiants en fin d´études de pharmacie en matière d´utilisation des antibiotiques.

## Conclusion

Cette étude a montré que la majorité des étudiants ont des connaissances suffisantes sur la RB et l´utilisation des antibiotiques. En revanche, leurs pratiques sont inadéquates dans la plupart des cas. Ces résultats montrent qu´il y a une nécessité de renforcer la réglementation et les programmes de formation sur l´usage rationnel des antibiotiques. La réalisation d´études qualitatives portant sur les raisons qui sous-tendent ces pratiques néfastes serait indispensable.

### Etat des connaissances sur le sujet

- *La consommation des antibiotiques est élevée dans le monde;*
- *La résistance aux antibiotiques est liée aux pratiques inadéquates en termes d´utilisation des antibiotiques;*
- *Les étudiants en pharmacie, en tant que futurs professionnels de la santé, ont un rôle important dans la lutte contre la résistance aux antibiotiques*.

### Contribution de notre étude à la connaissance

- *Les connaissances des étudiants en fin d´études de pharmacie sur les antibiotiques sont satisfaisantes;*
- *Les pratiques des étudiants en fin d´études de pharmacie en matière d´utilisation des antibiotiques sont inadéquates;*
- *Les étudiants ayant dans leur entourage un professionnel de la santé, ayant des connaissances suffisantes et étant en doctorat ont tendance à s´adonner aux pratiques inadéquates en matière d´utilisation des antibiotiques*.

## Remerciements

Nous remercions les étudiants en pharmacie qui ont accepté de participer à cette étude.
